# Citalopram Neuroendocrine Challenge Shows Altered Tryptophan and Kynurenine Metabolism in Migraine

**DOI:** 10.3390/cells11142258

**Published:** 2022-07-21

**Authors:** Kinga Gecse, Andrea Edit Édes, Tamás Nagy, Adrienn Katalin Demeter, Dávid Virág, Márton Király, Borbála Dalmadi Kiss, Krisztina Ludányi, Zsuzsanna Környei, Adam Denes, Gyorgy Bagdy, Gabriella Juhasz

**Affiliations:** 1 Department of Pharmacodynamics, Faculty of Pharmacy, Semmelweis University, Üllői Street 26, H-1085 Budapest, Hungary; 2 SE-NAP2 Genetic Brain Imaging Migraine Research Group, Hungarian Brain Research Program, Semmelweis University, Üllői Street 26, H-1085 Budapest, Hungary; 3 Department of Measurement and Information Systems, Faculty of Electrical Engineering and Informatics, Budapest University of Technology and Economics, Magyar Tudósok krt.2, H-1521 Budapest, Hungary; 4 Department of Pharmaceutics, Faculty of Pharmacy, Semmelweis University, Üllői Street 26, H-1085 Budapest, Hungary; 5 Momentum Laboratory of Neuroimmunology, Institute of Experimental Medicine, Szigony Street 43, H-1083 Budapest, Hungary; 6 NAP-2-SE New Antidepressant Target Research Group, Hungarian Brain Research Program, Semmelweis University, Üllői Street 26, H-1085 Budapest, Hungary; 7 MTA-SE Neuropsychopharmacology and Neurochemistry Research Group, Hungarian Brain Research Program, Semmelweis University, Üllői Street 26, H-1085 Budapest, Hungary

**Keywords:** headache, stress, biomarker, RANTES, neuroendocrine challenge, cytokine

## Abstract

Altered tryptophan (TRP) metabolism may have an important role in migraine susceptibility through its main metabolites, serotonin and kynurenine (KYN). Both affect pain processing and stress response by interfering with neural and brain hypersensitivity and by interacting with chemokines and cytokines that control vascular and inflammatory processes. The involvement of these pathways in migraine has been widely studied, but acute citalopram neuroendocrine challenge on TRP metabolism and cytokine profile has not been investigated yet. In our study, females with episodic migraine without aura and healthy controls were studied before and after acute citalopram or placebo in a double-blind setting. At baseline, increased TRP/large neutral amino acid (LNAA) ratio and decreased RANTES chemokine concentration were detected in migraine patients compared to controls. The challenge induced a significant increase in TRP, KYN, and TRP/LNAA in healthy controls, but not in migraine patients. Furthermore, migraine attack frequency negatively correlated with KYN/TRP ratio and positively correlated with the neuroendocrine-challenge-induced KYN concentration increase. Our results support a decreased breakdown of TRP via KYN pathway and a failure to modulate TRP–KYN pathway during citalopram-induced acute stress together with an increased vascular sensitivity in migraine. These mechanisms may provide useful drug targets for future drug development.

## 1. Introduction

Migraine is considered the primary cause of disability in young women, despite the advances in diagnosis and treatment in recent decades [1]. Different theories of migraine have been developed over the years, but nowadays migraine is described as a neurovascular disorder accompanied by altered brain sensory processing [2]. The involvement of serotonin in migraine pathophysiology was among the first to be discovered [3,4]. The observation of increased sensitivity to alterations in serotonergic neurotransmission in migraine patients has led to the development of the first migraine-specific drugs, called triptans [5], which act not only as vasoconstrictors as initially thought, but also through neural mechanisms by inhibiting trigeminal afferents and central trigeminal neurons [6]. Despite their effectiveness [7], we still do not know who will respond to triptan treatment, but there are promising studies to find markers of responsiveness [8]. In spite of the fact that potential cardiac side effects related to triptans directed drug discovery to other targets, serotonin remains in sight of migraine research [9].

Since serotonin does not cross the blood–brain barrier, dietary intake and plasma concentration of L-tryptophan (TRP), its essential precursor, affect brain serotonin synthesis [10] and potentially migraine susceptibility. TRP uptake to the brain is dependent on plasma concentration of other large neutral amino acids (LNAAs), as they compete for a common transporter [11]. The ratio of TRP/LNAA is widely used to describe the portion of TRP uptake to the brain; however, plasma TRP/LNAA ratio in migraine has not been investigated yet. Studies concerning the blood TRP concentration of migraine patients are not consistent; both higher [12,13,14,15] and lower [16,17] TRP concentrations were reported in migraineurs compared to controls. Furthermore, migraine patients are suggested to be sensitive to TRP concentration changes; thus, TRP depletion induces intense headache, nausea, and photophobia in migraineurs [18]. In addition, consuming relatively less TRP increases the risk for developing migraine in susceptible persons [19]. However, TRP supplementation alone has not proved to be an effective anti-migraine therapy [20,21].

One potential explanation of the uncertain therapeutic effect of TRP in migraine could be related to its complex metabolism. Although a small fraction of TRP serves serotonin synthesis, the main metabolic pathway of TRP is the kynurenine (KYN) pathway, which is also known to be involved in migraine [14,17,22,23]. Ninety-five percent of TRP is metabolized into N-formyl-kynurenine via two enzymes: tryptophan-2,3-dioxygenase (TDO) in the liver or indolamine-2,3-dioxyganese (IDO) in peripheral tissues and the nervous system [10]. After this rate-limiting step, L-kynurenine (KYN) is formed by formamidase enzyme. KYN is the main component of this pathway, as several active metabolites are synthesized from KYN, such as the neuroprotective kynurenic acid (KYNA) and the excitotoxic quinolinic acid (QUINA) [10].

The KYN–KYNA pathway may exert its effect on migraine via influencing glutamatergic neurotransmission. In migraine, glutamate acting on N-methyl-D-aspartate (NMDA) receptors induces the dilation of intra- and extracranial vessels [24] and also has an important modulatory effect on the descending pain control system in the brainstem, which influences trigeminal activation and nociception [2]. As KYNA is an endogenous NMDA receptor antagonist, it exerts an antinociceptive effect in the trigeminovascular system and may be a possible target of anti-migraine therapy [25,26]. In previous studies, decreased plasma KYN concentration was reported in both episodic [17] and chronic [14] migraine patients in interictal period compared to controls, suggesting a metabolic imbalance in TRP pathways of migraineurs [22]. KYN concentration is directly proportional to TRP concentration, and the KYN/TRP ratio is widely used to represent the shift of TRP metabolism from serotonin synthesis to KYN pathway [27]; previously, it was used as an indicator of IDO activity [28]. Even though the involvement of KYN pathway in migraine is well-known, there is still no anti-migraine therapeutic agent in the market acting on this pathway.

Serotonin and KYN may also have a notable role in migraine pathophysiology due to their regulatory influence on inflammatory processes [22,29]. KYN contributes to chemotactic activation of peripheral monocytes and also has a potential neuroimmunoregulatory role [28,30], while accumulating evidence suggests that alteration of the serotonin level in the body by serotonin reuptake inhibitors (SSRIs) modulates immune cell function and cytokine production [31]. However, the exact role of inflammatory processes in migraine pathophysiology is intensely debated [32]. The sterile neurogenic inflammation is hypothesized to contribute to the development of migraine attacks [33]. During a migraine attack, the activation of trigeminal neurons induces neuropeptide release; among others, calcitonin gene-related peptide (CGRP) [34,35] is a successful drug target for novel antimigraine therapies [36], leading to increased vascular permeability, vasodilation, and sensitization of the trigeminal neurons [37]. Furthermore, recent in vitro studies suggest that CGRP may increase the production of pro-inflammatory mediators, among them IL-1β, IL-6, and TNF-α, in the trigeminal ganglia [38]. Cytokines could act as pain mediators in neurovascular inflammation and play a role in pain generation in migraine [39]. The increased level of chemokines reinforces the stimulation of trigeminal neurons and thus the release of vasoactive peptides [40]. In addition, pro-inflammatory cytokines shift the metabolism of tryptophan toward the KYN pathway by activating the IDO enzyme [41]. Nevertheless, the investigation of cytokines in human migraine provided conflicting results, suggesting a possible increase in serum IL-1β, IL-6, and TNF-α levels in migraineurs compared to controls [42], depending on the ictal or interictal phase [32].

To further investigate the role of TRP metabolism and cytokine production in migraine and to identify potential migraine biomarkers, we investigated the baseline plasma concentration of tryptophan pathway and cytokine profile in interictal migraine patients without aura compared to healthy controls at two time-points at least two weeks apart in order to determine trait-like alterations. In addition, we examined serotonergic responsivity using a low-dose acute citalopram challenge, which is a safe neuroendocrine probe [43,44]. Citalopram is the most selective serotonin-reuptake inhibitor (SSRI) and increases extracellular brain serotonin availability without binding to any receptor type, and it also acts peripherally on serotonin-transporter-expressing cells. Intravenous administration of the drug is favored to avoid pharmacokinetic issues [43]. Acute citalopram challenge induces an increased arousal-like state [45] and a neuroendocrine stress response by increasing prolactin, ACTH, and cortisol release by stimulating the hypothalamic–pituitary–adrenal (HPA) axis [46,47]. Therefore, it is used as a neuroendocrine challenge to examine acute stress response [43,46,47]. Our previous study demonstrated that migraine patients are more sensitive to citalopram challenge showing increased brain activation in the anterior cingulate cortex compared to controls, for which observation suggests that increased stress-sensitivity in migraineurs is partially mediated by the serotonergic system [48]. However, to the best of our knowledge, the neuroendocrine response of migraine patients in acute citalopram challenge has not been investigated yet, nor has this challenge been used to determine the alterations of tryptophan metabolites and cytokines during acute serotonin level increase. Our additional aim was to investigate the correlation between clinical parameters of migraine and plasma concentration of tryptophan pathway and cytokine profile in baseline condition and after acute citalopram neuroendocrine challenge.

## 2. Materials and Methods

### 2.1. Study Design

**Figure 1:**
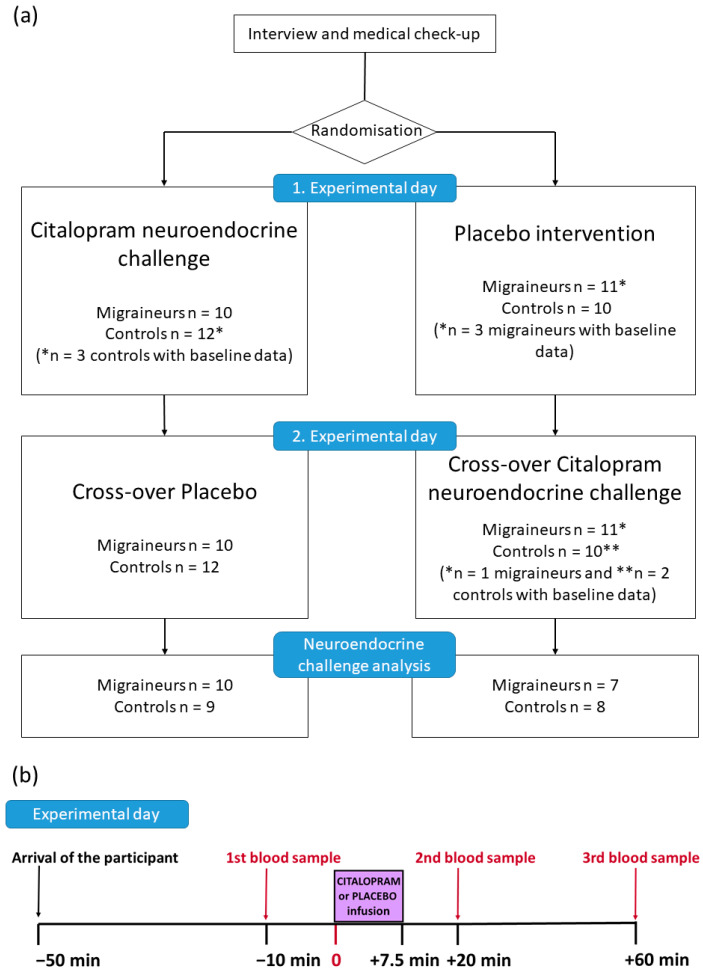
The study design. (**a**) Overall design of the randomized, cross-over study. Participants with only baseline data had missing variables; therefore, we could not include them in neuroendocrine challenge analysis. They were only included in baseline data analysis. (**b**) The design of each experimental day.

Participants received 7.5 mg citalopram infusion in a randomized, double-blind crossover design. Normal saline or citalopram was infused over 7.5 min. The experimental days were separated by at least two weeks to evaluate them as independent sessions. In previous studies, 7.5 mg dose intravenous citalopram was proven to be an effective neuroendocrine challenge. Blood samples were collected 10 min before the infusion as well as 20 and 60 min after the infusion. The first blood sample was collected around 50 min after the participants’ arrival to let them acclimatize and to reduce the effect of experiment-induced stress (Figure 1).

Every five minutes, participants were asked about their subjective experiences, and they answered with yes/no for the following statements: anxious, nauseous, drowsy, lightheaded, restless, uncomfortable.

The experiments were conducted between 4:00 pm and 8:00 pm in order to avoid the impact of circadian variations in cortisol and DHEA-S concentration [49,50].

### 2.2. Participants

Forty-three female participants were included in the study: 21 patients with episodic migraine without aura and 22 healthy controls, between 18–50 years of age. The recruitment was based on university advertisements and newspaper articles, and some migraineurs volunteered from Headache Clinics. The participants underwent a diagnostic interview and medical screening, and their mental health status was checked using the Mini-International Neuropsychiatric Interview [51]. Exclusion criteria were the following: having any past or current serious medical, neurological (except migraine without aura), or psychiatric disorders; use of any daily medication (except contraceptives). All participants refrained from alcohol for at least 24 h and caffeine for at least 4 h before taking the first blood sample. In the case of four migraineurs and five controls, there were technical problems in the blood sample collection during the citalopram neuroendocrine challenge. Their data were included in the baseline concentration analysis but excluded from the neuroendocrine challenge analyses. Among the included participants, 17 migraineurs and 17 controls successfully completed the study without missing data and were included in the neuroendocrine challenge analyses.

### 2.3. Clinical Variables of Migraine

The diagnosis of episodic migraine without aura was given by expert neurologists based on the International Classification of Headache Disorders-III criteria [52]. Migraine with aura patients were excluded from the study. Participants were pain- and medication-free 48 h before and 24 h after the examination days and they were not taking any preventive migraine medications. They were asked to provide information about the clinical indicators of migraine such as age at disease onset and frequency of attacks per month.

### 2.4. Ethics

The study was conducted in accordance with the Declaration of Helsinki. Each participant was informed regarding the aim of the study, the substance used, and possible aversive side effects, and they provided written informed consent. The study protocol was approved by the Scientific and Research Ethics Committee of the Medical Research Council, Hungary (number: 23609-1/2011-EKU, 23421-1/2015-EKU).

### 2.5. Biological Samples

The blood samples were collected into K3EDTA tubes. After immediate centrifugation (10 min at 2000 RPM), plasma samples were kept frozen at −80 °C until the assay. For the quantitative determination of tryptophan, kynurenine, and other large amino acid (valine, leucine, isoleucine, phenylalanine, and tyrosine) concentration, LC–MS/MS method was used. The detailed methodology was published by Virág et al. [53]. Plasma cortisol and dehydroepiandrosterone sulfate (DHEA-S) levels were measured by competitive ELISA kits from NovaTec Immunodiagnostica GmbH (Dietzenbach, Germany) according to the manufacturer’s instructions [54]. The coefficient of variation expressing inter- and intra-assay variance were as follows: cortisol, 11.0% and 5.1%; DHEA-S, 10.4% and 7.9%, respectively. The cortisol antibody showed 46.2% cross-reaction with prednisolone, while the DHEA-S antibody showed cross-reaction with DHEA (100%) and androstenedione (59%).

The concentrations of cytokines and the main chemokines induced by them [55,56] (granulocyte colony-stimulating factor (GCSF), interleukin 1 alpha (IL1a), interleukin 1 beta (IL1b), interleukin 6 (IL6), interleukin 8 (IL8), interleukin 10 (IL10), tumor necrosis factor alpha (TNFa), monocyte chemoattractant protein-1 (MCP-1), and regulated upon activation, normal T-cell-expressed, and presumably secreted CCL5 (RANTES)) were determined by BD Cytometric Bead Array (CBA) using BD CBA Flex Sets according to the manufacturer’s instructions. Samples were acquired using a BD FACSVerse flow cytometer (BD, Becton, Dickinson and Company, Franklin Lakes, NJ, USA), and the results were analyzed by FCAP Array software (BD, Becton, Dickinson and Company, Franklin Lakes, NJ, USA) [57]. Average blanks for the cytokines and chemokines were the following: GCSF: 2.57 pg/mL, IL1a: 2.38 pg/mL, IL1b: 0 pg/mL, IL6: 0 pg/mL, IL8: 0 pg/mL, IL10: 2.89 pg/mL, MCP-1: 0.58 pg/mL, RANTES: 7.46 pg/mL, and TNFa: 1.40 pg/mL.

The concentration of plasma citalopram was measured with liquid-chromatography-tandem mass spectrometry (Agilent 1260 Infinity LC system, Agilent Technologies, Santa Clara, CA, USA).

### 2.6. Statistical Methods

Statistical analysis of the data was conducted in SPSS (SPSS Statistics for Windows, Version 27.0, IBM Corp., Armonk, NY, USA) Mann–Whitney tests were used to determine any differences between migraine and control groups regarding age and baseline plasma concentrations. The sum of LNAA affecting tryptophan blood–brain barrier crossing (namely tyrosine, phenylalanine, leucine, isoleucine, and valine) was calculated after Fernstrom et al. [11]. The ratio of tryptophan and LNAA (TRP/LNAA), the ratio of kynurenine and tryptophan (KYN/TRP), and the ratio of cortisol (CORT) and DHEA-S (CORT/DHEA-S) were calculated. The ratio of CORT/DHEA-S was used as an index of the neurobiological stress response [58]. Considering the non-normally distributed data, we used the Friedman test to determine the effect of the citalopram neuroendocrine challenge on tryptophan pathway and inflammatory markers in all participants and in separately analyzing the control and migraine group. For detected significant differences in plasma concentration, post-hoc analysis with Wilcoxon signed-rank tests was applied with a Bonferroni correction. Kruskal–Wallis test was applied to investigate the difference in citalopram plasma concentrations between migraine and control groups. The age at migraine onset and migraine frequency were correlated with baseline plasma concentrations and the neuroendocrine-challenge-induced concentration changes (calculated as the difference of the 20-min, 60-min, and baseline concentrations) using Spearman correlations. The significance threshold was set at *p* < 0.05. Although our sample size was relatively low, previous studies suggested that we had sufficient power to detect the effect of the citalopram neuroendocrine challenge [43,44,46,47,59,60,61].

For data visualization, GraphPad Prism version 8.0.1 for Windows software (GraphPad Software, San Diego, CA, USA) was used.

A partial least-squares linear discriminant analysis (PLS-LDA) model [62] was established to pattern discrimination between migraine patients and healthy controls [17]. PLS is a potent dimension-reduction method that is applicable in scenarios with multicollinearity where the measure of relevance for individual variables is also important. Two PLS models were established, each producing three latent variables as output: (1) the baseline model, containing biological markers from the blood samples before the citalopram infusion, and (2) the neuroendocrine challenge model, containing markers from blood samples taken 20 min after citalopram administration. Il1b was not included in the PLS-LDA model, because it was 0 pg/mL for every participant. The latent variables resulting from the PLS step were used in an LDA classifier to test the diagnostic capabilities of the models. Because of the small amount of data, we used leave-one-out cross-validation to test the predictive power of the full model without overfitting. The contribution of each variable to the latent variables of the PLS model was described with the variable importance projection (VIP) score, where values above 1.0 are indicative of relevance. The PLSRegression and LinearDiscriminantAnalysis modules of the sklearn package (version 1.0.2) were used in Python 3.8.5 (Python Software Foundation, Wilmington, DE, USA).

## 3. Results

### 3.1. Descriptive Characteristics of the Participants

There was no significant difference in age between the two groups (migraine: 25.00 (23.00 to 28.00) (median years (95% CI)), control: 24.50 (23.00 to 27.00) (median years (95% CI)); U = 236.5, *p* = 0.893). The mean age at migraine onset was 15.48 ± 6.23 (mean years ± SD). The mean of migraine attack frequency was 3.57 ± 3.29 (mean attacks per month ± SD).

### 3.2. Baseline Plasma Concentrations

Both citalopram and placebo infusion were preceded by blood sampling to determine the baseline concentration of tryptophan (TRP), kynurenine (KYN), cortisol (CORT), and the ratio of TRP/LNAA, KYN/TRP, CORT/DHEA-S, and the concentration of inflammatory cytokines and chemokines (GCSF, RANTES, MCP-1, IL1a, Il1b, IL6, IL8, IL10, and TNFa) before any intervention (Table 1).

The TRP/LNAA ratio was significantly higher in the migraine group compared to controls at both blood samplings. The TRP concentration was not significantly different between migraine patients and controls, but it tended to be higher in migraineurs at both blood-sampling occasions. The RANTES concentration was lower in the migraine group compared to controls on both experiment days. The IL8 concentration was higher in the migraine group compared to the control before placebo infusion; however, there was no difference between the two groups before citalopram intervention. There were no further differences between migraine patients and controls at baseline.

### 3.3. Citalopram Neuroendocrine Challenge

#### 3.3.1. Main Effect of Citalopram Neuroendocrine Challenge

**Figure 2:**
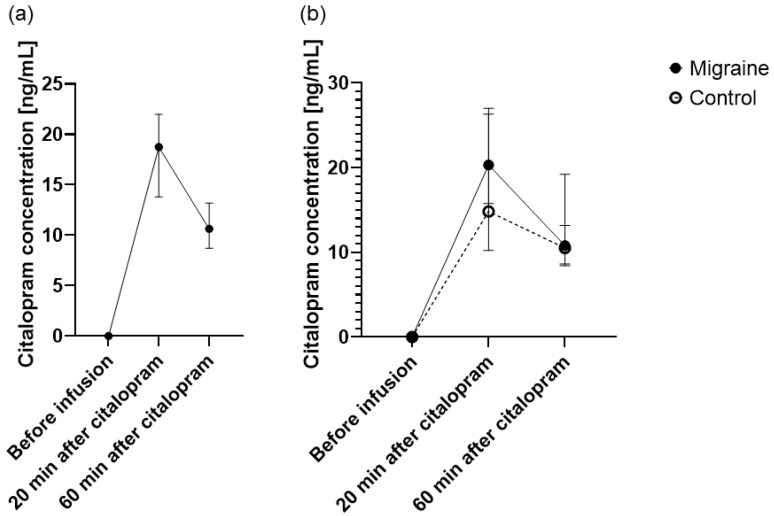
Effect of citalopram neuroendocrine challenge on plasma citalopram concentration. The median plasma concentration of citalopram (±95% CI) 20 min and 60 min after infusion (**a**) in the whole population and (**b**) separately in the migraine and control groups.

The citalopram neuroendocrine challenge significantly increased the plasma citalopram level in the whole study population (20 min: median [ng/mL] (95% CI) = 18.74 (13.81 to 21.98); 60 min: median [ng/mL] (95% CI) = 10.62 (8.71 to 13.18)) (Figure 2a), and the plasma concentrations corresponded well with previous studies [43,63]. Post hoc analysis showed that there was no significant difference in plasma citalopram concentration between migraine (20 min: median [ng/mL] (95% CI) = 20.32 (15.76 to 26.32); 60 min: median [ng/mL] (95% CI) = 10.79 (8.42 to 13.18)) and control (20 min: median [ng/mL] (95% CI) = 14.82 (10.21 to 27.00); 60 min: median [ng/mL] (95% CI) = 10.51 (8.61 to 19.20)) groups (20 min: H(1) = 2.75, *p* = 0.097; 60 min: H(1) = 0.15, *p* = 0.904) (Figure 2b).

Administration of IV citalopram significantly increased the concentration of plasma TRP (χ2(2) = 6.35, *p* = 0.042), KYN (χ2(2) = 11.53, *p* = 0.003), RANTES (χ2(2) = 11.12, *p* = 0.004) and TRP/LNAA ratio (χ2(2) = 6.59, *p* = 0.037) in the population. During the placebo condition, CORT concentration (χ2(2) = 31.83, *p* < 0.001) and CORT/DHEA-S ratio (χ2(2) = 26.65, *p* < 0.001) showed significant time-dependent decrease that cannot be seen after the citalopram neuroendocrine challenge.

#### 3.3.2. Differences between the Effects of Citalopram Neuroendocrine Challenge in Migraine Patients and Controls

There were no differences between the migraine and the control groups regarding the subjective experiences during the citalopram neuroendocrine challenge. Of the migraine patients, 12.5% (2/16) (one migraine patient did not answer) and 5.89% (1/17) of controls reported anxiety (*p* = 0.601), 18.75% of migraine patients and 23.53% of controls reported nausea (*p* = 1.000), 68.75 % of migraine patients and 58.82% of controls reported drowsiness (*p* = 0.721), 25% of migraine patients and 23.53% of controls reported dizziness (*p* = 1.000), 31.25% of migraine patients and 29.41% of controls reported restlessness (*p* = 1.000), and 56.25% of migraine patients and 41.18% of controls reported discomfort (*p* = 0.494) as a side-effect during the citalopram neuroendocrine challenge.

**Figure 3:**
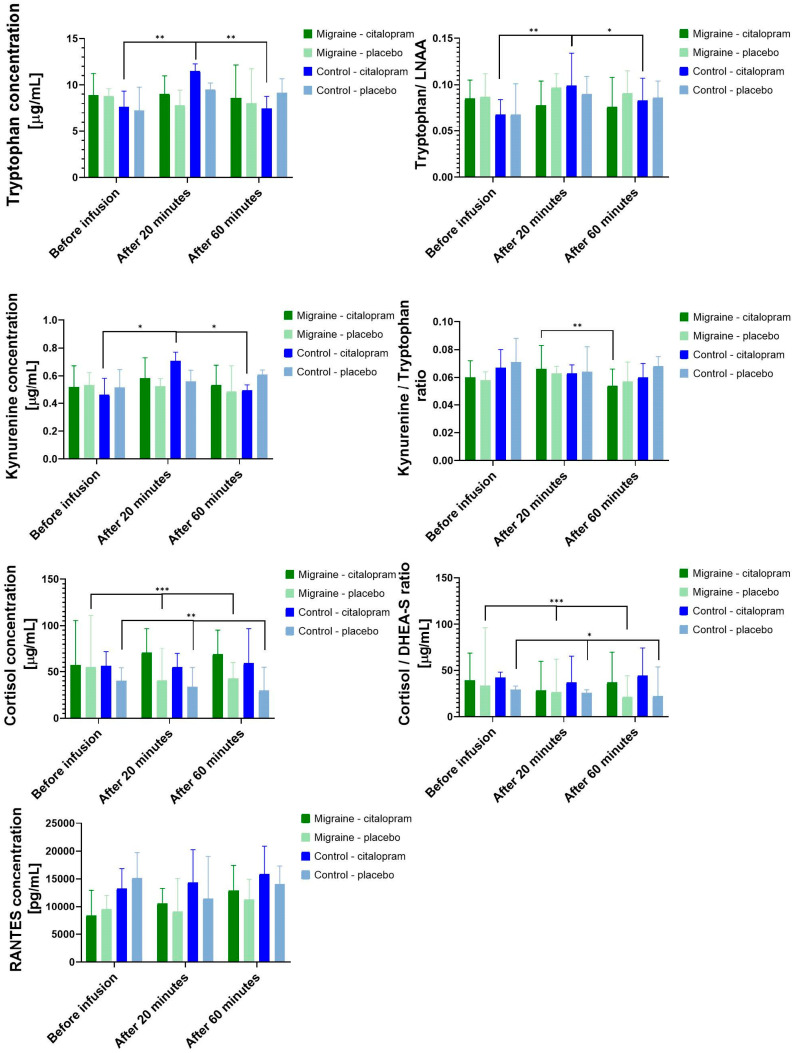
Significant effect of citalopram neuroendocrine challenge on the measured biomarkers. Medians and 95% confidence interval for tryptophan, kynurenine, cortisol, and RANTES plasma concentrations and tryptophan/LNAA, kynurenine/tryptophan, and cortisol/DHEA-S ratios. LNAA: large neutral amino acids, DHEA-S: dehydroepiandrosterone sulfate * *p* < 0.05, ** *p* < 0.01, *** *p* < 0.001.

The citalopram neuroendocrine challenge showed a diagnosis-dependent effect on TRP, KYN concentration and on TRP/LNAA, KYN/TRP ratios (Figure 3). Separately analyzing the control group, we found that the citalopram neuroendocrine challenge induced significant increase in the TRP (χ2(2) = 14.94, *p* < 0.001), KYN (χ2(2) = 8.94, *p* = 0.011) concentration and TRP/LNAA ratio (χ2(2) = 13.18, *p* < 0.001). There was a significant time-dependent increase in the TRP (χ2(2) = 7.18, *p* = 0.028) concentration of control subjects after placebo, although the magnitude of change was lower compared to the citalopram neuroendocrine challenge. In the migraine group, the KYN/TRP ratio (χ2(2) = 7.41, *p* = 0.025) decreased significantly after the citalopram neuroendocrine challenge, and no other significant change could be detected regarding the TRP pathway. The CORT concentration (migraine: χ2(2) = 23.48, *p* < 0.001; control: χ2(2) = 9.88, *p* = 0.007) and CORT/DHEA-S ratio (migraine: χ2(2) = 20.24, *p* < 0.001; control: χ2(2) = 7.88, *p* = 0.019) showed the expected circadian decrease in both groups during placebo condition, but this time-dependent change was not observed in the neuroendocrine challenge condition. As to the cytokines and chemokines, the changes of RANTES concentration after the neuroendocrine challenge showed no difference between the migraine (χ2(2) = 5.77, *p* = 0.056) and the control groups (χ2(2) = 5.77, *p* = 0.056) (Figure 3).

In the control group, there were no significant differences after post-hoc analysis between baseline and 60-min values of TRP concentration (Z = 0.06, *p* = 1.000) after citalopram. However, there was a statistically significant elevation of TRP concentration 20 min after citalopram infusion compared to baseline (Z = −1.12, *p* = 0.003) and a significant reduction in TRP concentration 60 min later compared to the blood sample taken after 20 min (Z = 1.18, *p* = 0.002).

Regarding the KYN concentration changes in the control group, there were no significant differences between the baseline and the citalopram concentration of KYN (Z = 0.12, *p* = 1.000) after 60 min. However, there was a statistically significant elevation of KYN concentration 20 min after citalopram infusion compared to baseline (Z = −0.82, *p* = 0.049) and a significant reduction in 60 min KYN concentration compared to the blood sample taken after 20 min (Z = 0.94, *p* = 0.018).

In the control group, there were no significant differences between the baseline and the citalopram ratio of TRP/LNAA (Z = −0.24, *p* = 1.000) after 60 min. However, there was a statistically significant elevation of TRP/LNAA ratio 20 min after citalopram infusion compared to baseline (Z = −1.18, *p* = 0.002) and a significant reduction of TRP/LNAA ratio after 60 min compared to the blood sample taken after 20 min (Z = 0.94, *p* = 0.018).

Post-hoc test of the KYN/TRP ratio changes in the migraine group showed no significant difference between the baseline and the 20-min KYN/TRP (Z = −0.71, *p* = 0.119) and between the baseline and the 60-min KYN/TRP ratio (Z = 0.18, *p* = 1.000). However, a significant reduction was observed between the 20-min KYN/TRP ratio and the 0 min KYN/TRP ratio (Z = 0.88, *p* = 0.003).

### 3.4. The Relationship of Migraine Parameters with the Measured Biomarkers

**Figure 4:**
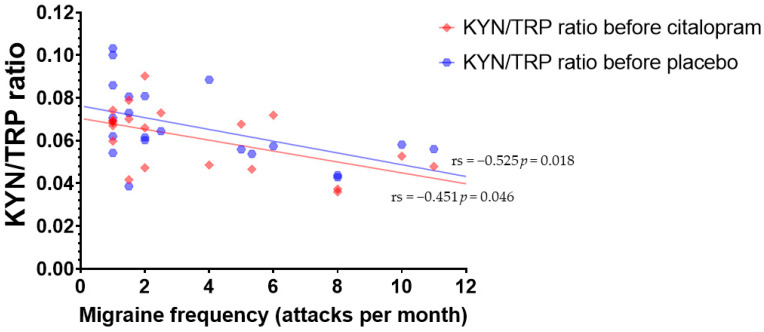
Correlation between number of migraine attacks per month and the ratio of kynurenine (KYN) and tryptophan (TRP) before citalopram neuroendocrine challenge (r_s_ = −0.451 *p* = 0.046) and placebo (r_s_ = −0.525 *p* = 0.018) infusions.

The frequency of migraine attacks was negatively correlated with KYN/TRP ratio before citalopram neuroendocrine challenge (r_s_ = −0.451 *p* = 0.046) and placebo (r_s_ = −0.525 *p* = 0.018) infusions (Figure 4).

**Figure 5:**
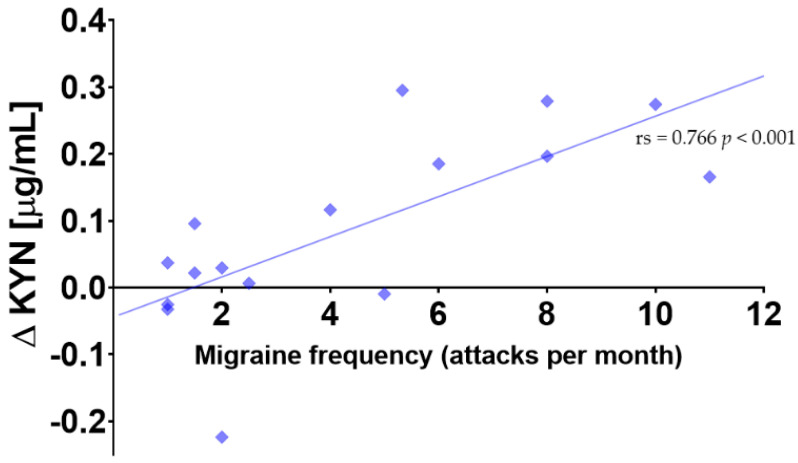
Correlation between number of migraine attacks per month and citalopram neuroendocrine-challenge-induced kynurenine (KYN) concentration changes (r_s_ = 0.766 *p* < 0.001). ДKYN = The difference between the concentrations measured at 20 min after citalopram infusion started and baseline.

The citalopram neuroendocrine-challenge-induced concentration changes of the plasma KYN 20 min after the start of the infusion showed positive correlation with the frequency of migraine attacks (r_s_ = 0.766 *p* < 0.001) (Figure 5).

There were no significant associations between other biomarkers and migraine frequency or age at migraine onset.

### 3.5. Discriminating Migraine Patients from Controls with PLS-LDA

**Figure 6:**
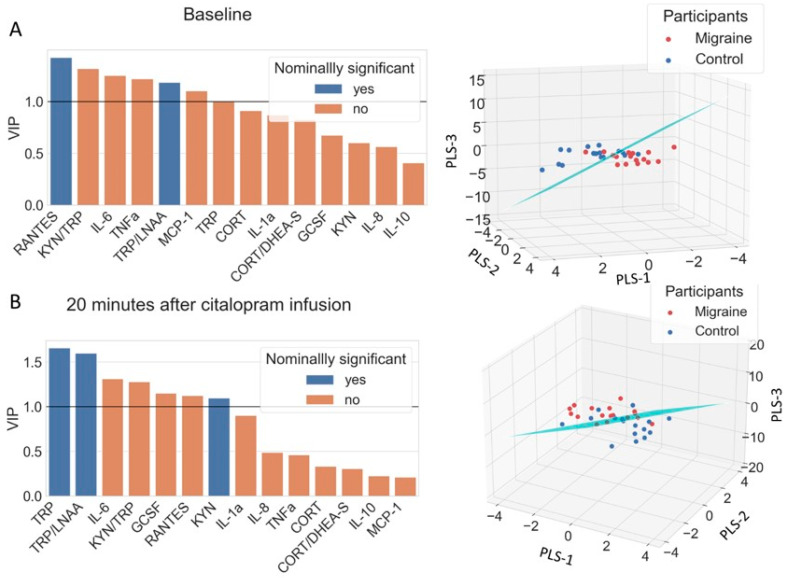
The bar plots of partial least-squares linear discriminant analysis (PLS-LDA) and 3D projection plots. (**A**) Baseline model representing the biological data before any intervention. The variable importance projection scores (VIP) above 1 indicates relevance in migraine. (**B**) Citalopram neuroendocrine challenge model representing the biological data 20 min after citalopram infusion. The classification hyperplane distinguishes the migraine group compared to controls. TRP: tryptophan, LNAA: large neutral amino acids, KYN: kynurenine, CORT: cortisol, DHEA-S: dehydroepiandrosterone sulfate, GCSF: granulocyte colony-stimulating factor, RANTES: regulated upon activation, normal T-cell-expressed and presumably secreted CCL5, MCP-1: monocyte chemoattractant protein-1, IL1a: interleukin 1 alpha, IL1b: interleukin 1 beta, IL6: interleukin 6, IL8: interleukin 8, IL10: interleukin 10, TNFa: tumor necrosis factor alpha.

The multivariate classification method was used to identify the variables that play a role in the differentiation between migraine and control group. The baseline model contained the biological data before any intervention (Figure 6A). RANTES, TRP/LNAA, KYN/TRP, IL6, TNFa, and MCP-1 showed greater VIP score than 1, indicating relevance in migraine diagnosis. The citalopram neuroendocrine challenge model used the biological data 20 min after citalopram infusion (Figure 6B). In that case, TRP, TRP/LNAA ratio, KYN, IL6, KYN/TRP ratio, GCSF, and RANTES showed greater VIP score than 1. The resulting diagnostic accuracies of the full model were 79% for the baseline model and 71% for the citalopram model.

## 4. Discussion

According to our knowledge, this is the first study about TRP pathway and cytokine profiles of episodic migraine without aura patients before and after the citalopram neuroendocrine challenge. Trait-like alterations were identified in migraine patients, namely increased TRP/LNAA ratio and decreased RANTES concentration, compared to controls in two independent baseline plasma samples. The acute neuroendocrine citalopram challenge that can also reflect stress-responsivity showed significant differences between migraine patients compared to controls. More precisely, the citalopram neuroendocrine challenge induced the increase of TRP and KYN concentration and TRP/LNAA ratio in healthy controls, but not in migraine patients. Furthermore, the frequency of migraine attacks showed negative correlation with baseline KYN/TRP ratio, another trait-like marker in two independent blood samples, but it positively correlated with the citalopram neuroendocrine-challenge-induced KYN concentration increase. Thus, our study supports a conclusion that the downregulation of the TRP–KYN pathway, probably partially through a maladaptive stress response, may contribute to migraine pathophysiology.

### 4.1. Alterations of the TRP Metabolism in Migraine

Increased plasma TRP/LNAA ratio was detected in interictal episodic migraine without aura patients compared to healthy controls at two independent blood samplings. To the best of our knowledge, our study is the first investigation of plasma TRP/LNAA ratio in migraine patients, as previous studies only reported results related to plasma TRP concentration [12,13,14,15,16,17,64]. In this study, the TRP concentration was not significantly higher in the migraine group, but it tended to be higher compared to controls at both blood samplings. Our results are consistent with previous studies reporting elevated TRP concentration in episodic [12,15] and chronic migraine patients [13,14]. However, there are some studies reporting the opposite results [16,17]. The opposing results may be due to different study populations and different time points for blood sampling. In our study, only patients with episodic migraine without aura who do not take prophylactic medication were included, and samples were collected in the afternoon, in contrast to other studies where samples were collected from headache clinic patients in the morning [17], and they were allowed to use prophylactic drugs [14,17]. Plasma TRP concentration shows changes with circadian rhythm, so the time of blood sampling could have an effect on the results [65]. Moreover, TRP uptake into the brain is affected by plasma LNAA concentration [11]; thus, plasma TRP concentration might not be an adequate biomarker in migraine. However, the TRP/LNAA ratio is a reliable marker of TRP brain uptake [11].

One possible explanation of the increased plasma TRP/LNAA ratio could be the decreased breakdown of TRP due to the impairment of TDO and IDO enzyme activity in migraine [22]. TDO and IDO enzymes are the rate-limiting step of KYN formation from TRP [10]. TDO is expressed in the liver, and astrocytes in the brain, while IDO is expressed in peripheral tissues, immune system cells, and microglia [66]. In a nitroglycerin administration rodent model, Nagy-Grócz et al. [67] demonstrated that migraine may be related to decreased expression of KYN pathway enzymes (kynurenine amino transferase-II (KAT-II), IDO, TDO, KYNU, KMO). Thus, our results could be a further support to the notion of downregulation of KYN enzymes in migraine.

The elevated TRP/LNAA ratio suggests an increased TRP intake by interictal migraine brain [12,14,64]. The increased TRP availability might be a protective factor between migraine attacks providing adequate TRP level for brain serotonin synthesis. TRP depletion studies demonstrated that diminished brain TRP level through decreased serotonin synthesis provokes intense headache, nausea, and photophobia in migraine patients [18]. Furthermore, relatively less TRP consumption increases the risk for developing migraine in susceptible people [19]. However, in our previous study, we did not find any correlation between migraine indicators (attack frequency and age at onset) and plasma TRP concentration, but an association between TRP concentration with depressive symptoms and trait-anxiety was apparent in migraine patients [15], which in turn may affect the recurrence of attacks and the processing of pain stimuli. Moreover, the increased plasma TRP concentration (corrected for plasma LNAA concentration) was associated with decreased functional connectivity of periaqueductal gray matter with regions implicated in fear-cascade and pain processing but increased functional connectivity with frontal emotion and pain regulating areas, thereby supporting TRP having an important role in optimizing stress coping [15]. Our previous results might suggest that serotonergic pathways synthetized from TRP could be more responsible for emotional symptoms and stress-processing in migraine than for the attack generation itself. Indeed, in the present study, the KYN pathway showed association with migraine frequency, as the decreased KYN/TRP ratio negatively correlated with the increasing number of migraine attacks per month at two independent blood samplings. Previously, the ratio of KYN/TRP was used to measure IDO activity; however, the current view is that the KYN/TRP ratio is more suitable to demonstrate the TRP pathway shift from serotonin to KYN [27]. Therefore, our results further suggest a less active kynurenine pathway in the background of migraine.

### 4.2. Migraine Patients Failed to Activate TRP Pathway during Citalopram Neuroendocrine Challenge

This is the first study reporting plasma TRP pathway and cytokine profile alterations during citalopram neuroendocrine challenge in humans. Acute citalopram challenge is a neuroendocrine probe to test serotonergic responsivity, and it induces acute stress [43]. We detected an immediate elevation of plasma TRP, KYN and TRP/LNAA ratio in healthy controls during the citalopram neuroendocrine challenge, but not in migraine patients, supporting the role of maladaptive stress-responsivity in migraine [68,69].

Acute stress increases the plasma concentration of TRP and KYN via different processes [70]. Previous studies showed that activation of the HPA axis increases plasma amino acid levels [71]; however, in our study, plasma TRP elevation preceded the significant increase in cortisol level. Thus, a more plausible explanation for our results is that the activation of the sympathetic nervous system may contribute to TRP increase [72]. The increase in the TRP/LNAA ratio suggests that the brain influx of TRP is also increased during the short-term effects of citalopram. Only free, non-protein-bound TRP can enter the brain [11]. The majority of plasma TRP is bound to circulating albumin and competes for its binding site with non-esterified fatty acids (NEFA) or drugs [71]. Elevation of NEFA level could happen under several circumstances, such as upon activation of sympathetic nervous system during physical exercise [73] or during neuroendocrine challenge, and this could displace TRP from protein binding [10,71], eventually leading to increased TRP brain uptake, which promotes serotonin synthesis and stress-coping [74].

The increase in TRP and KYN, without a change in the KYN/TRP ratio, could also represent an acute increase of the KYN pathway activity, in which induction of IDO and TDO enzymes by citalopram neuroendocrine challenge may play a role [75,76]. Indeed, acute physiological and psychological stress, such as neuroendocrine stress, could also induce KYN pathway enzymes [77]. TDO is mainly induced by cortisol, IDO induced by cytokines, and these processes enhance brain input of KYN for further metabolic transformations [66]. Based on animal experiments and our results, the intricate balance between TRP and KYN metabolites in healthy controls may contribute to stress resilience [78]. However, our results also suggest that these changes are temporary in healthy controls because the short-term increase in TRP and KYN concentration was followed by a rapid decrease 60 min after citalopram infusion, presumably due to the activation of the TRP and KYN degradation enzymes [78].

In migraine patients, TRP and TRP/LNAA plasma concentration did not increase from the citalopram neuroendocrine challenge. A likely explanation for this observation is that the already-elevated TRP concentration in migraine patients, which might be necessary to sustain the interictal phase or could be a sign of increased stress sensitivity [15], could not be elevated further by the acute activation of the sympathetic nervous system. Moreover, the induction of IDO and TDO enzymes may also be less effective, since enzymes of the KYN pathway are expressed in a reduced manner in migraine, resulting only in a modest increase in KYN plasma level [67]. Therefore, the dampened response of the TRYP–KYN pathway in migraineurs for the citalopram neuroendocrine stress challenge further emphasizes that the downregulated TRYP–KYN pathway may have a pathophysiologic role in migraine.

Interestingly, KYN concentration did not increase significantly in migraine patients during the citalopram neuroendocrine challenge, but the magnitude of the increase was associated with migraine frequency. Although we did not measure it in our study, previous research suggested that KAT expression is lower in migraine patients; thus, KYN conversion to KYNA is diminished [79]. KYNA has an important antinociceptive effect both in the periphery and in the brain [22]; by blocking first-order neurons and CGRP release [80], it could also reduce the activation of serotonergic neurons in the raphe nuclei [81] and have analgesic effect when injected directly into the PAG [82]. The decreased KYNA production could cause increased NMDA receptor hyperactivity in migraine that leads to brain hyperexcitability [66]. Therefore, despite increased plasma TRP level interictally, the insufficiently increased plasma KYN concentration during acute stress and the downregulated KYN metabolic pathway may lead to higher migraine attack frequency.

### 4.3. Inflammatory Biomarkers in Migraine

Although the neurogenic inflammation theory of migraine has been debated intensely [2], cytokines and chemokines may contribute to the development of migraine attacks [33], and they may have a role in migraine chronification [32]. Our classical statistical analysis showed that our subjects have no systemic inflammation based on their low IL-6, Il-1, and TNFa cytokine concentrations; furthermore, no significant difference appeared in plasma cytokines (namely GCSF, IL1a, IL1b, IL6, IL10, and TNFa) between migraineurs and healthy controls in the interictal phase. The plasma concentration of three chemokines was also determined in this study, namely MCP-1, RANTES, and IL8. Higher IL8 concentration was found in migraine patients compared to controls, but this was not replicated at the second blood sampling. However, decreased RANTES plasma concentration in migraine patients was replicated at both blood samplings. Furthermore, the multivariate analysis of our data suggested that RANTES, IL6, TNFa, MCP-1, and GCSF, together with the TRP–KYN pathway, might also be important in shaping the vulnerability to migraine by modulating inflammatory and vascular processes [83].

In recent decades, RANTES has gained more attention in headache research since Fidan et al. [84] demonstrated that RANTES levels rise during migraine attacks, but they found no difference between attacks in migraine patients compared to controls. Similarly, another study showed that the expression of RANTES gene (*CCL5*) is upregulated during migraine attacks, contributing to platelet activation [85]. Regarding other cytokines and chemokines, our results are in concordance with Fidan et al.’s [84] study, because they also reported no difference in IL10 and MCP-1 concentration between interictal migraineurs and healthy controls. Sarchielli et al. [86] examined the chemokine levels in jugular venous blood of migraine patients during attacks, but they did not find any difference in MCP-1 and RANTES concentration between the ictal and interictal state. They involved only eight migraine patients in the study, so the low number of participants may explain the contradictory findings. Domingues et. al. [87] reported an increased RANTES concentration in migraine patients compared to tension-type headache patients and suggested that increased anxiety and depressive symptoms of migraine patients could explain the difference. In our study, both migraine and control participants were free from any mental disorders, including anxiety or depression diagnosis. This is one possible explanation for our results differing from previous studies where this was not an exclusion criterion; the other is that we investigated the concentration of cytokines and chemokines in attack-free periods of migraineurs.

On the basis of the neurovascular theory of migraine pathophysiology, the hyperexcitability of trigeminovascular system is the key point of migraine attacks, although the nature of the primary triggers is still debated [2]. The trigeminal neurons innervate the dural blood vessels and contribute to the release of neuroinflammatory mediators parallel with the activation of the trigeminal system, which could be responsible for migraine pain and allodynia, as the release of the neuroinflammatory mediators is accompanied by dilation of the dural blood vessels [2]. Although vasodilation alone is not sufficient to initiate migraine attacks [88], accumulating evidence suggests that different vascular mechanisms are instrumental in migraine pathophysiology. For example, in previous studies, lower RANTES concentrations were associated with higher flow-mediated dilation (FMD) in high cardiovascular risk people [89], and higher FMD was observed in migraineurs with aura compared to migraineurs without aura and controls [90]. However, another study found no difference in FMD between migraine and non-migraine individuals [91]. In our study, episodic migraine without aura patients had decreased plasma RANTES concentration compared to healthy controls at two independent blood samplings. Contrary to previous studies, our results suggest that low RANTES concentration, presumably through an increased vascular sensitivity to blood-flow-induced dilation [89], may also contribute to episodic migraine without aura, which is in line with the neurovascular hypothesis of migraine. The low RANTES level is also consistent with GWAS studies of migraine, in which genes related to the vascular system are over-represented in migraine patients, but vascular genetic risk factors that showed an association with migraine did not increase the risk of hypertension or cardiovascular diseases [92]. Thus, our results, together with the previous findings, suggest that decreased RANTES concentration may contribute to interictal vascular hypersensitivity in migraine patients, predisposing them for migraine attacks, and the ictally increased RANTES concentration might be associated with the platelet activation that was observed during attacks.

In addition, we detected an increase in RANTES concentration during the citalopram neuroendocrine challenge both in migraine and control participants. Acute citalopram administration by inducing a neuroendocrine response leads to an acute stress reaction [43,47]. Our results suggest that during a neuroendocrine stress condition, RANTES concentration rises independently from migraine diagnosis. Increased RANTES is suggested to play a role in dysfunctional vascular regulation by the modulation of perivascular inflammation [89,93], so the increasing RANTES concentration might be a part of the vascular response in neuroendocrine stress. However, in our study, RANTES concentration remained lower in migraine patients (median: 10,580 pg/mL (13,292–5882) 95% CI) compared to controls (median: 14,340 pg/mL (20,233–7260) 95% CI), even during the neuroendocrine stress challenge. However, the difference was not significant, suggesting a permanent, trait-like RANTES-related increased vascular sensitivity in migraine patients, which supports the vascular component of neurovascular migraine theory [2]. The exact role of RANTES in migraine generation is still unknown; however, our results suggest that it might act through pro-inflammatory, vascular, and nociceptive effects.

### 4.4. Limitations

Our study has some important limitations. The first is the relatively low number of participants involved in the analysis, which is partially due to the complexity of the neuroendocrine citalopram challenge paradigm. However, the randomized, double-blind crossover design (Figure 1) and the availability of two independent baseline samples per participant strengthen our results. The second limitation of our study is the fact that we measured plasma total TRP concentration and did not investigate the free and bound TRP ratio. However, we determined plasma LNAA concentrations because the TRP/LNAA ratio is a much better marker for the TRP influx into the brain. Finally, not measuring the expression and the activity of the KYN pathway enzymes and the plasma concentration of other KYN metabolites is another limitation of our study. Instead, we focused on chemokines and cytokines beside TRP, KYN, and LNAA in order to capture the effect of the citalopram neuroendocrine challenge in migraineurs not only on the KYN pathway but also on neuroinflammation. Indeed, our multivariate analysis supports the role of these biological systems in migraine vulnerability.

## 5. Conclusions

We have provided further evidence for KYN pathway downregulation in migraine through trait-like decrease in TRP and KYN metabolism. There is increasing evidence that a decreased amount of KYN pathway metabolites plays an important role in migraine pathogenesis affecting glutamate signaling. Our results also demonstrated an increased susceptibility to vascular changes, indicated by decreased RANTES concentration in migraine patients at trait level in interictal period. Furthermore, an altered response of KYN pathway was observed in migraineurs during the citalopram neuroendocrine challenge, which distinguishes migraine patients from controls. Further studies are needed to investigate whether migraine prevention is possible by influencing the TRP–KYN pathway. In ongoing clinical trials, glutamate receptor (mGLUR5) modulators and AMPA/kainite receptor antagonists are promising treatments for acute migraine, as are NMDA and kainate receptor antagonists for migraine prophylaxis. These targets are under the influence of KYN pathway metabolites.

## Figures and Tables

**Table 1:** Baseline concentrations of the measured biomarkers before citalopram neuroendocrine challenge and placebo infusions.

	Before Citalopram Neuroendocrine Challenge		*p*-Value	Before Placebo Infusion		*p*-Value
	Migraine	Control		Migraine	Control	
*Tryptophan, kynurenine(µg/mL), cortisol (ng/mL) and cortisol/DHEA-S (µg/mL)*						
TRP	8.92 (6.75–10.18)	7.12 (5.55–9.28)	0.075	8.20 (6.85–9.47)	7.15 (5.59–8.11)	0.080
TRP/LNAA	0.09 (0.06–0.11)	0.06 (0.05–0.08)	**0.012**	0.08 (0.07–0.11)	0.06 (0.05–0.09)	**0.033**
KYN	0.57 (0.40–0.67)	0.46 (0.39–0.54)	0.308	0.54 (0.46–0.62)	0.47 (0.39–0.64)	0.274
KYN/TRP	0.07 (0.05–0.07))	0.07 (0.06–0.07)	0.264	0.06 (0.06 to 0.08)	0.07 (0.06–0.08)	0.481
CORT	58.74 (40.23–118.0)	56.72 (38.15–80.86)	0.512	64.21 (51.13–111.0)	40.86 (31.23–66.54)	0.382
CORT/DHEA-S	46.49 (37.62–85.32)	42.10 (33.41–58.25)	0.662	34.48 (35.86–82.30)	28.88 (23.90–53.65)	0.423
*Cytokines and chemokines (pg/mL)*						
GCSF	4.19 (3.64–4.83)	3.95 (3.54–4.91)	0.894	4.18 (3.94–4.94)	4.18 (3.39–5.13)	0.423
RANTES	10641 (6336–11,588)	13456 (10,192–16,846)	**0.017**	10593 (7279–12,588)	12670 (10,091–19,710)	**0.049**
MCP-1	15.01 (10.70–29.94)	13.96 (8.70–19.99)	0.538	16.94 (9.24–32.08)	15.74 (8.32–22.04)	0.388
IL1a	2.69 (2.20–3.62)	2.42 (1.92–3.00)	0.224	2.96 (2.22–3.11)	2.85 (1.36–3.47)	0.696
IL1b	0 (0.00–0.00)	0 (0.00–0.00)	1.000	0 (0.00–0.00)	0 (0.00–0.00)	1.000
IL6	0 (0.00–0.00)	0 (0.00–0.00)	0.163	0 (0.00–0.00)	0 (0.00–0.00)	0.143
IL8	0 (0.00–7.85)	0 (0.00–6.81)	0.748	7.13 (0.00–8.67)	0 (0.00–0.00)	**0.014**
IL10	3.20 (3.14–3.39)	3.26 (3.14–3.46)	0.473	3.46 (3.32–3.61)	3.27 (3.11–3.60)	0.193
TNFa	1.96 (1.50–2.20)	1.41 (0.89–2.40)	0.233	1.96 (0.79–2.22)	1.26 (0.66–2.22)	0.752

Note: Values demonstrate median ±95% confidence intervals; Mann–Whitney U test was used to compare the plasma concentrations between migraine and control groups with a *p* < 0.05 significance threshold (bold). TRP: tryptophan, LNAA: large neutral amino acids, KYN: kynurenine, CORT: cortisol, DHEA-S: dehydroepiandrosterone sulfate, GCSF: granulocyte colony-stimulating factor, RANTES: regulated upon activation, normal T-cell-expressed, and presumably secreted CCL5, MCP-1: monocyte chemoattractant protein-1, IL1a: interleukin 1 alpha, IL1b: interleukin 1 beta, IL6: interleukin 6, IL8: interleukin 8, IL10: interleukin 10, TNFa: tumor necrosis factor alpha.

## Data Availability

The datasets generated and analyzed during the current study are not publicly available due to ongoing analysis for future publication, but are available from the corresponding author upon reasonable request.
